# Identifying components of a theory-informed complex intervention supporting primary care nurses to provide evidence-based dementia care in the general practice setting

**DOI:** 10.1017/S1463423626101674

**Published:** 2026-09-07

**Authors:** Caroline Gibson, Mark Yates, Dianne Goeman, Dimity Pond, Jill J. Francis

**Affiliations:** 1 The University of Newcastle - Newcastle Callaghan Campus: University of Newcastle, School of Medicine and Public Health, Australia; 2 Deakin University School of Medicine, Australia; 3 Wicking Dementia Research and Teaching Centre, University of Tasmania, Australia; 4 Faculty of Medicine Dentistry and Health Sciences, The University of Melbourne, Australia

**Keywords:** behaviour change theory, complex intervention, dementia, nurses, practice change, primary care, theorical domains framework

## Abstract

**Aim:**

To demonstrate the application of the Theoretical Domains Framework (TDF) and behaviour change theory to identify behaviour change techniques for a complex intervention targeting dementia care by general practice nurses (GPNs).

**Background:**

GPNs have an important role in dementia care but require support to translate evidence-based recommendations into routine practice. Theory-informed implementation strategies may help facilitate this change.

**Methods:**

The target behaviour was derived from a Delphi study identifying national dementia care recommendations relevant to GPNs. Barriers to operationalizing the target behaviour were identified from previous research and then coded independently by two authors using the TDF. Identified domains were then mapped to behaviour change techniques using the Theory and Techniques Tool.

**Findings:**

The specified behaviour was: ‘the general practice nurse will use language that is accurate, respectful, inclusive, empowering and non-stigmatizing when communicating with people living with dementia within each patient encounter in general practice’. Seven TDF domains were identified, including ‘knowledge’, ‘skills’ and ‘social influences’, mapping to 22 behaviour change techniques, including ‘instruction on how to perform the behaviour’ and ‘social support’. This theory-informed approach identifies practical techniques for developing interventions to improve GPN communication with people living with dementia and provides a method that could be applied to other dementia care behaviours in general practice.

## Introduction

Increasingly, global health policy promotes a more proactive approach to the diagnosis and management of dementia in the primary care setting (Prince *et al.,* 2016; Sideman *et al.,* 2023; Fowler *et al.,* 2025). However, it is well established that there are multiple barriers to the identification, diagnosis and management of dementia in primary care (Fox *et al.,* 2013; Mansfield *et al.,* 2018; Arsenault-Lapierre *et al.,* 2022; Gibson *et al.,* 2023). This suggests changes in the organization, and delivery of care are needed to improve the accessibility and quality of care for people living with dementia.

Nurses working in general practice, hereafter referred to as general practice nurses (GPNs), are significant contributors in the delivery of primary care worldwide (Norful *et al.,* 2017). Mobilizing an existing workforce has the potential to reduce the time and cost needed to meet the health care needs of people living with dementia as opposed to creating a new specialist workforce (Prince *et al.,* 2016). GPNs already play a role in chronic disease management (Stephen *et al.,* 2018). Given that co-morbidity in people living with dementia is high (Bunn *et al.,* 2014), the GPN is likely to have an established therapeutic relationship with people experiencing cognitive impairment. GPNs themselves also perceive that they have a role in dementia care provision (Trickey *et al.,* 2000; Gibson *et al.,* 2021; White *et al.,* 2024) and people living with dementia and their informal carers agree (Gibson *et al.,* 2024a). A recent Australian study identified five recommendations contained in the Clinical Practice Guidelines and Principles of Care for People with Dementia (Guidelines Adaptation Committee, 2016) relevant to the provision of best-practice dementia care by the GPN (Gibson *et al.,* 2023). These recommendations emphasized the delivery of person-centred care as vital for effective assessment and care planning for the person living with dementia (Gibson *et al.,* 2023). Person-centred dementia care is universally accepted as best practice care for people living with dementia (Kitwood, 1997; Sunjaya *et al.,* 2025). Central to person-centred care is the establishment of therapeutic relationships which maintain personhood. Personhood is defined as the quality or condition of being a person regardless of cognitive ability (Fazio *et al.,* 2018).

For GPNs to successfully operationalize the dementia care recommendations in their daily practice, effective implementation strategies to support a change in clinical practice are needed (Fischer *et al.,* 2016; Karrer *et al.,* 2020). To achieve such changes, passive dissemination of guidelines is rarely sufficient (Grol *et al.,* 2020). A wealth of evidence shows that more active strategies, or ‘implementation interventions’, are required. Given the multi-faceted needs of people living with dementia, and the complexity of health systems in which they are managed, such implementation interventions will be complex. The UK Medical Research Council recommends the use of theory in the development and evaluation of complex interventions that aim to facilitate behaviour change (Skivington *et al.,* 2021b).

The Theoretical Domains Framework (TDF) was developed to identify the barriers and enablers of behaviour change also referred to as determinants of change (Cane *et al.,* 2012; Michie *et al.,* 2015; Atkins *et al.,* 2017). The TDF comprises 14 theoretical domains: ‘knowledge’, ‘skills’, ‘social/professional role and identity’, ‘beliefs about capabilities’, ‘optimism’, ‘beliefs about consequences’, ‘reinforcement’, ‘intentions’, ‘goals’, ‘memory, attention and decision processes’, ‘environmental context and resources’, ‘social influences’, ‘emotion’ and ‘behavioural regulation’ (Cane *et al.,* 2012). Each theoretical domain describes the mechanisms of action that can influence health practitioner behaviour. The TDF has been successfully used in the development of implementation interventions in healthcare settings including primary care (Taylor *et al.,* 2013; Porcheret *et al.,* 2014; Cadogan *et al.,* 2016; Debono *et al.,* 2017; Courtenay *et al.,* 2019; Lim *et al.,* 2020; Dyson and Cowdell, 2021; Guldhammer *et al.,* 2023). In the context of this paper, an implementation intervention is defined as ‘a method or technique designed to enhance the adoption’ of a healthcare intervention (Curran *et al.,* 2012).

Once the determinants of change related to the desired behaviour are identified, they can be mapped to Behaviour Change Techniques (BCT) as defined in the Behaviour Change Technique Taxonomy (BCTTv.1) (Michie *et al.,* 2013). BCTs are defined as the smallest identifiable, reproducible elements of an intervention that can cause a change in behaviour (Michie *et al.,* 2015). The application of the BCTTv.1 provides a standardized process to design and describe the reproducible content of a behaviour change intervention (French *et al.,* 2012).

This paper describes the systematic application of the TDF to interpret primary qualitative research findings and inform intervention development by identifying BCTs that could address problematic domains (or barriers). For the purposes of demonstrating this process we have selected one clinical behaviour drawn from the five best-practice recommendations relevant to the provision of dementia care by the GPN (Gibson *et al.,* 2023). The barriers and enablers to the clinical behaviour were drawn from previous research in dementia care provision by GPNs (Gibson *et al.,* 2024b). The outcome of this paper is the identification of BCTs to consider in the development of a theory-informed complex intervention to change GPN behaviour in the provision of best-practice dementia care. To our knowledge this approach has not previously been used to target an intervention aimed at GPNs’ provision of dementia care in their clinical practice.

### Study aims

The aims of this study were to use behaviour change theory to:

1. Select and specify the target behaviour for change (who should do what differently?)
2. Identify the theoretical domains that encompass the barriers and enablers to the target behaviour by GPNs (which factors make it more difficult or less difficult to perform these target behaviours?)
3. Identify the BCT that address the identified domains and that therefore can be considered in the development of a theory-informed intervention to support GPNs provide care to people living with dementia (which techniques could support GPNs to change how they do these things?)

## Methods

**Figure 1.**
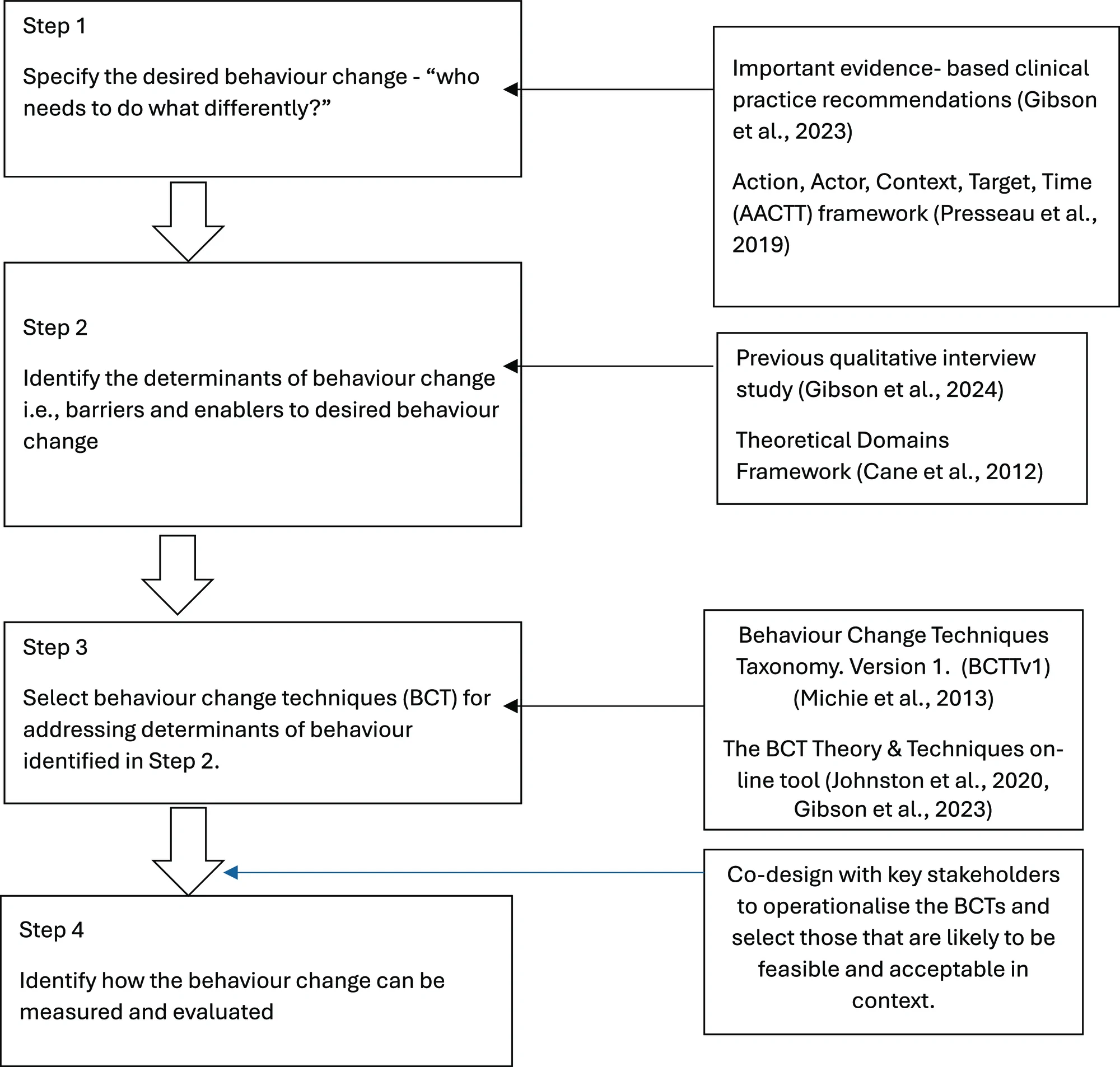
Process used in the identification of a theory-informed complex intervention components based on French *et al.* (2012).

We employed the first three steps of the French *et al.* (2012) four-step systematic method to guide the identification of potential components of a complex intervention. This systematic method uses guiding questions to direct the choice of the most appropriate components of an implementation intervention (French *et al.,* 2012) (Figure 1). The fourth step (co-design and measurement) is to be addressed in a planned randomized control study to evaluate the effectiveness of the proposed implementation intervention.

### Step 1: select and specify the target behaviour for change

#### Select the target behaviour

**Figure 2.**
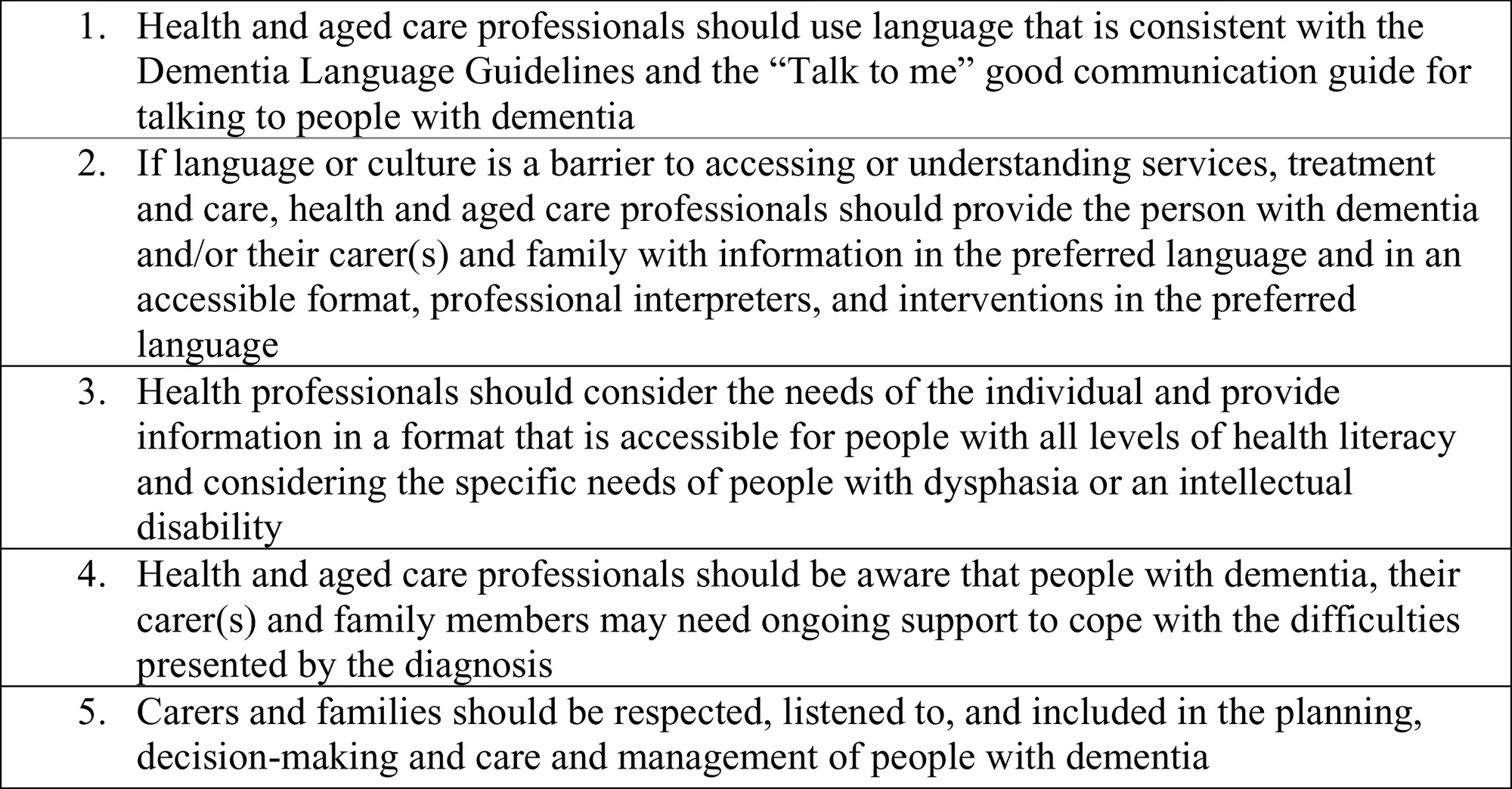
Practice recommendations highly relevant to the clinical care of people living with dementia by the general practice nurse (Gibson *et al*., 2023).

The first step was to identify the target clinical behaviour, (who should do what differently) based on best-practice recommendations developed to address the evidence-practice gap in dementia care. A recent Delphi study (Gibson *et al.,* 2023) identified five recommendations in the Clinical Practice Guidelines and Principles of Care for People with Dementia (Guidelines Adaptation Committee, 2016) highly relevant to the clinical care of people living with dementia by the GPN. These recommendations are described in Figure 2.

To demonstrate the theoretically informed identification of potential components of a complex intervention, we used the first of these five recommendations as an example throughout this paper. This recommendation is ‘Health and aged care professionals should use language that is consistent with the Dementia Language Guidelines and the “Talk to me” good communication guide for talking to people with dementia’, hereafter referred to as ‘good communication’.

We selected this recommendation because ‘good communication’ is a clinical behaviour performed by GPNs, potentially modifiable at practitioner level, and is observable and measurable. Communication between healthcare practitioners and those they care for is a key element of a person-centred care approach to dementia care (Kitwood, 1997; Sunjaya *et al.,* 2025) and was central to all five recommendations.

#### Specify target behaviour

To provide an operational definition of the selected recommendation it was necessary to identify the key behavioural components. Dementia Australia developed both the Dementia Language Guidelines (Dementia Australia, 2023) and the ‘Talk to me’ (now called ‘Let’s Talk’) (Dementia Australia Advisory Committee, 2024) good communication guide. These two documents are included within this recommendation as informing what constitutes ‘good communication’ with people living with dementia. Using the descriptions provided in these two documents, two authors (CG and MY) used a consensus process to describe the target behaviour of good communication based as having five components. These components are described in Table 1.

**Table 1.** Key components of the target behaviour of ‘good communication’ Table 1 long description.

As described in the Dementia Language Guidelines, in their communication with people living with dementia GPNs should-	Description of the behaviour component described in the ‘Talk to me’ (now called Let’s Talk’) communication guide
Be accurate	Learn the facts about dementia Avoid using jargon, as I might misunderstand.
Be respectful	Understand what dementia means to me Use language preferred by me
Be inclusive	Please talk with me, not my carer, family member or friend. Make a connection with me. Make eye contact. Use my name so I know you’re talking to me. Make sure I am listening.
Be empowering	Reduce noise and other distractions to help me to focus. Use short sentences, with one idea at a time. Be patient and understanding. Don’t rush me. Give me more time to respond. Give me time to ask questions.
Be non-stigmatizing	Do not use demeaning and derogatory terms to describe people living with dementia. Use language that recognizes me as a person and retains my feelings of self-worth.

After selecting the target behaviour, a detailed specification of the behaviour(s) targeted for change is required. This is to ensure the intervention components and measured outcomes align in the implementation of the intervention (Atkins *et al.,* 2017; Presseau *et al.,* 2019). We used the Action, Actor, Context, Target, Time (AACTT) framework to systematically specify the desired behaviour change (Presseau *et al.,* 2019). The AACTT framework enables consistent description, specification and monitoring of clinical behaviours. (Presseau *et al.,* 2019). In this model, action describes the clinical act, the actor is the person who does the clinical act, the context is where the act is carried out, the target is the person with, for, or for whose benefit, the action is performed, and the time is when the clinical act occurs. Time may encompass frequency and duration when appropriate.

### Step 2: identify the theoretical domains that encompass the barriers and enablers to the target behaviour by GPNs

This step identifies which factors make it more difficult or less difficult to perform the target behaviours. A previous qualitative study was conducted with GPNs to identify self-perceived barriers and enablers to the implementation of the five recommendations in their clinical practice (Gibson *et al.,* 2024b) (Refer Table 2.). Barriers to the GPN operationalizing the recommendation on ‘good communication’ included a lack of knowledge about dementia, reluctance to ask about cognition, competing priorities and time constraints. Enablers to ‘good communication’ included conducting conversations in environments where patients felt comfortable and building trust in nurse-patient relationships.

**Table 2.** General practice nurse barriers and enablers to operationalizing ‘good communication’ (Gibson *et al.,* 2024b) Table 2 long description.

Behaviour to operationalize selected practice recommendation	Identified barriers/enablers (determinants of behaviour change) to operationalizing this recommendation from previous study (BLINDED)
Be accurate when talking about dementia	GPNs want dementia to be documented by the GP in the patient record before they feel they can have a direct conversation about dementia with a patient and or family
	Poor knowledge about dementia presentation and impact is a barrier to good communication
Be respectful of the person’s understanding of dementia	A lack of understanding of the different cultural contexts of dementia can be a barrier to good communication
	A lack of communication skills can be a barrier, for example, skills needed to adjust language to meet the person’s level of understanding as dementia progresses
Be inclusive and allow people the opportunity to speak for themselves	Patients trust and feel safe with nurses and will talk with them about their concerns [enabler]
	Competing priorities limit time during the consultation to allow the person living with dementia to process information and respond to questions
Be empowering by providing a comfortable relaxed environment	GPNs perceive busy clinic environments are noisy and distracting so they try to get person living with dementia out quickly, so the patient does not get agitated
	Nurses perceive home visits result in more informative conversations as the person living with dementia and their carer are in a familiar environment [enabler]
Be non-stigmatizing and use positive language when talking about dementia	A lack in GPN confidence talking about dementia when it is perceived as a scary topic of conversation
	Reluctance to ask questions about dementia because patients and or family may become upset

We next coded the barriers and enablers to the TDF to identify domains to target for behaviour change. A table describing the TDF domains was used as the coding framework (Table 3). Two authors (CG, a primary care nurse clinician with experience in qualitative dementia research and JF, an expert in behavioural approaches to implementation science in healthcare) deductively coded the barriers and enablers to the TDF domains. Barriers and enablers could be coded into more than one domain. The coders then met to discuss the coding outcomes. Any discrepancies were resolved via discussion, with each coder providing an explanation for why data was assigned or not to a domain. A third author (MY, dementia care researcher familiar with the TDF) was consulted if agreement was not reached.

**Table 3.** Coding framework describing TDF domains and definitions adapted from Cane *et al.* (2012) Table 3 long description.

TDF domains	Definitions (with examples contextualized for this study)
Knowledge	An awareness of the existence of the Dementia Language Guidelines. This can include knowledge about dementia presentation and impact or ‘procedural’ knowledge (about the key elements of how to communicate well).
Skills	The ability to communicate well with a person living with dementia, acquired through practice. Interpersonal skills are included in this domain.
Social professional role and identity	Beliefs by the GPN that the patients trust nurses which facilitates patients disclosing cognitive concerns. This domain includes the social and professional role, identity and confidence of an individual in a social or work setting.
Beliefs about capabilities	Perceived competence and belief that an individual has in oneself. For example, GPNs perceived ability in good communication skills to complete a cognitive assessment tool correctly.
Optimism	Confidence that the GP will follow-up on the information GPNs gathering having a conversation about a patient’s cognitive concerns is an example of this domain.
Beliefs about consequences	A GPN belief that because there is no treatment for dementia there is no benefit of asking questions to assess cognition. This domain includes outcome expectancies and the potential for consequences including anticipated regret.
Reinforcement	Incentives, such as remuneration for longer consultations for the GPN to have the time needed to have difficult conversations about cognition and potential dementia with patients and families. Disincentives are also be included in this domain.
Intentions	A conscious decision to perform a behaviour or a resolve to act in a certain way. For example, deciding to discuss brain health and cognitive impairment during every care plan.
Goals	Setting targets, action planning and implementation intentions to achieve an outcome. For example, the GPN setting a goal to complete dementia and communication skills training within a certain timeframe.
Memory, Attention and Decision-making Process	Remembering to talk about cognition with patients would be an example of this domain which includes decision-making, cognitive attention and information retention.
Environmental context and resources	This domain can include built environment, organizational culture and resource availability that impacts on the development of skills, abilities and independence. An example of this domain may be a lack of organizational support for GPNs to attend education and training on dementia and communication skills.
Social influences	This domain includes power dynamics, hierarchy and social supports, norms and modelling that cause a person to change their thoughts, feelings or behaviours. GPN mentoring from nurses specialized in dementia and or communication skills could be an example of this domain.
Emotion	This domain includes emotional responses, including anxiety, fear, stress and burn-out impacting on a person performing a behaviour. For example, GPN may experience anxiety about upsetting patients if they talk about dementia.
Behavioural regulation	Self-reflection on outcomes of communication approaches, to identify what was successful and those that were not, could be included in this domain. Action-planning aimed at managing or changing objectively measured actions is in this domain.

### Step 3: identify the behaviour change techniques that address the identified domains

At the completion of step 2, a list of theoretical domains describing the determinants of change most likely to influence GPNs to perform the target behaviours was generated. To select the BCTs most likely to bring about the desired behaviour change we used the online Theory and Techniques Tool (Johnston *et al.,* 2020). This tool, based on published scientific literature and links agreed by experts in consensus exercises, provides a matrix linking BCTs to domains. Its use allows for a transparent, systematic, replicable and accessible method to link BCTs with the processes through which they change behaviour (Johnston *et al.,* 2020).

## Findings

### Selection and specification of the target behaviour for change

Using the AACTT framework (Presseau *et al.,* 2019) the target behaviour was specified as:

The general practice nurse (ACTOR) will use language that is accurate, respectful, inclusive, empowering and non-stigmatizing when communicating (ACTION) with people living with dementia (TARGET) within each patient encounter (TIME) in the general practice setting (CONTEXT).

### Identification of the theoretical domains that encompass the barriers and enablers to the target behaviour by GPNs

The barriers and enablers of behaviour change to the GPN operationalizing the recommendation on ‘good communication’ (Table 2) were deductively coded to the TDF domains using the definitions of each domain as described by Cane *et al.* (2012). The barriers and enablers were associated with seven of the 14 TDF domains as described in Table 4. For example, a barrier to ‘good communication’ was a lack in GPN confidence in talking about dementia. This was coded to the domain, ‘beliefs about capabilities’. An enabler to ‘good communication’ was trusted nurse-patient relationships. This was coded to the domain, ‘social influences’.

**Table 4.** Barriers and enablers to behaviour change and the theoretical domains to which they were coded Table 4 long description.

TDF	Barriers and enablers to the GPN performing the desired behaviour ‘The general practice nurse will use language that is accurate, respectful, inclusive, empowering and non-stigmatizing when communicating with people living with dementia within each patient encounter in the general practice setting’.
Social/professional role and identity AND Environmental context and Resources AND Social influences	GPNs want dementia to be documented by the GP in the patient record before they feel they can have a direct conversation about dementia with a patient and or family
Knowledge	Poor knowledge about dementia presentation and impact on the patient and carer(s) can be a barrier to good communication
Beliefs about Capabilities	A lack in GPN confidence talking about dementia when it is perceived as a scary topic of conversation by the nurse
Emotion	Reluctance to ask questions about dementia because patients and or family may become upset
Knowledge	A lack of understanding of dementia in different cultural contexts can be a barrier to good communication
Skills	A lack of communication skills needed to adjust language to meet the person’s level of understanding as dementia progresses
Social influences	Patients trust and feel safe with nurses and will talk with them about their concerns [enabler]
Environmental context and resources	Competing priorities limited time during the consultation to allow the person living with dementia to process information and respond to questions
Environmental context and resources AND Emotion	Busy clinic environments are noisy and distracting so they try to get person living with dementia out quickly so does not get agitated

### Identification of the behaviour change techniques that address the identified domains

Twenty-two BCTs were identified as the ‘active ingredients’ to include in an intervention targeting GPNs to change behaviour regards ‘good communication’ with people living with dementia. These BCTs are described in Table 5. For example, the physical environment could be restructured by setting up a quiet room for consultations with people living with dementia. The examples presented are only provided to illustrate the BCTs. Decisions about acceptable and feasible ways to operationalize the BCTs are the subject of a planned co-design study.

**Table 5.** Behaviour change techniques (BCTs) linked conclusively with determinants of change using the theory and techniques tool (Johnston *et al.,* 2020) Table 5 long description.

TDF domain (determinant of change)	Behaviour change technique (BCT)	An example of what the behaviour change technique could look like in the primary care context
Social/professional role and identity	No conclusive links identified between BCTs and determinants of change within this domain	
Environmental context and Resources	Social support (practical) Prompts/cues Remove aversive stimulus Restructuring the physical environment Restructuring the social environment Avoidance/ reducing exposure to cues for the behaviour Adding objects to the environment	Set up a quiet room for consultations with people living with dementia
Social influences	Social support (unspecified) Social support (practical) Social comparison Information about others approval Social reward	Mentoring by GPNs experienced in communicating with people living with dementia
Knowledge	Instruction on how to perform behaviour Information about antecedents Information about health consequences Information about social and environmental consequents	Provide Information on changes in communication experienced by people living with dementia.
Beliefs about capabilities	Problem-solving Instruction on how to perform behaviour Demonstration of behaviour Behavioural practice/rehearsal Graded tasks Verbal persuasion about capability Focus on past success Self-talk	Role play real-life clinic scenarios communicating with people living with dementia
Emotion	Reduce negative emotions	Provide information on the benefits of a dementia diagnosis and living well with dementia
Skills	Instruction on how to perform behaviour Behavioural practice/ rehearsal Graded tasks	Demonstrate good communication skills with people living with dementia.

## Discussion

There is evidence that the use of behaviour change theory can increase the success of interventions (Atkins *et al.,* 2017; Dyson and Cowdell, 2021). This approach has been used in the development of interventions targeting GPNs in a range of clinical behaviours, including cervical screening (Bravington *et al.,* 2022), increasing physical activity in diabetes (Avery *et al.,* 2015), self-harm prevention (Leather *et al.,* 2023), venous leg ulcer management (Weller *et al.,* 2020) and Human Papillomavirus related clinical care (McSherry *et al.,* 2012). To our knowledge this approach has not previously been used to target an intervention aimed at GPNs’ provision of dementia care in their clinical practice.

We identified ‘active ingredients’ to consider in the development of a theory-informed complex intervention to change GPN behaviour when communicating with people living with dementia. For the purposes of demonstrating this process, one clinical behaviour was selected from five best-practice recommendations identified as relevant to the provision of dementia care by the GPN (Gibson *et al.,* 2023). Good communication was selected as the clinical behaviour to focus on as it is key to the delivery of high-quality healthcare. Good communication is essential to establishing therapeutic relationships that support person-centred care (Sharkiya, 2023) which is the cornerstone of best-practice dementia care (Fazio *et al.,* 2018). Existing interventions promoting best-practice dementia care including communication skills have largely focussed on the delivery of education programmes targeting dementia knowledge and skills, particularly in residential care (Rasmussen *et al.,* 2023). While educational initiatives can increase knowledge, they are generally not sufficient to translate that knowledge into consistent behavioural changes in complex healthcare settings such as primary care (Lau *et al.,* 2015; Cadogan *et al.,* 2016). Multifaceted interventions targeting multiple barriers to change are more likely to be effective than single interventions.

This study supports the evidence that increasing health practitioner knowledge is a necessary but not sufficient step for behaviour change as additional determinants of change are also involved in supporting enhanced communication skills with people living with dementia. It is widely recognized that policy, professional and organizational contexts impact on the design and implementation of complex interventions (Skivington *et al.,* 2021a). The development of an intervention to support GPNs to change their behaviour in relation to communication with people living with dementia is no exception. One barrier to ‘good communication’ was described by GPNs as it not being their place or position to be first to bring up topic of cognition if the GP has not first discussed it with the patient. Reluctance to bringing up the topic of cognitive impairment may reflect the wider social stigma surrounding dementia and cognitive impairment and perceived harm and or anticipated regret (Visser *et al.,* 2024). However, this barrier was also reflected within a hierarchical primary care structure where nurses work under the direction of the GP. Australian primary care is funded by GP activity. This means that the nurse only generates income if working under the GP’s direction and carrying out delegated tasks that generate income. This funding model limits the nurse providing proactive care (Gibson *et al.,* 2024b). High-performing primary care encompasses a team-based approach (Ghorob and Bodenheimer, 2015) and GPNs who have collaborative, respectful working relationships with the GP are more likely to be proactive in the care they provide and work to the scope of their practice (McInnes *et al.,* 2017). Wanting the GP to document a cognitive impairment before GPNs feel able to bring up the topic presents a significant barrier to GPNs providing appropriate care to people living with dementia and their carer(s), since the poor documentation of dementia by GPs has been well established (Davis *et al.,* 2021). This is a complex barrier and problematic for a GPN-focussed intervention because they have very little control over what a GP writes in their notes. In the cultural workplace environment of the primary care clinic, it is also difficult for GPNs to act in isolation. Therefore, it may not be feasible to address this barrier immediately with a GPN focussed intervention as a whole-practice approach may be required.

This study provides an illustration of methods for identifying BCTs that may be associated with intervention effectiveness for enhancing communication with people living with dementia. Actively collaborating with key stakeholders in intervention development increases the potential for effective design, implementation and dissemination of innovations in practice (Vargas *et al.,* 2022). Hence, the next steps are a co-design study with GPNs to identify which of the potential BCTs identified in this study are acceptable and feasible in an intervention, and how they might best be operationalized. This would be followed by a randomized controlled trial to address the empirical question as to whether an intervention designed using this robust process leads to measurable effectiveness.

## Strengths and limitations

A strength of this study is the systematic application of behaviour change theory to interpret primary qualitative research findings and inform intervention development. The barriers and enablers to communication were drawn from studies with Australian nurses which could limit the international generalizability of interventions developed. Data were coded retrospectively using the TDF domains. While this allowed for identification of the issues that are most salient to the research question, using the TDF to inform data collection may have elicited more in-depth information on barriers that were not reported in the larger study.

## Conclusion

With rising demand for better dementia care in the primary care setting, these findings will guide interventions that support appropriate dementia care behaviours among GPNs. Using behaviour change theory to identify BCT enhances the likelihood that implementation interventions will have an impact on clinical practice. This structured, transparent approach also supports clear reporting, enables replication across settings, and promotes the use of reproducible components shown to change behaviour.

## Data Availability

No new data were generated or analysed in support of this research.

## Table 1. Long description

A table comparing guidelines for communicating with people living with dementia. The table has five rows and two columns. The first column lists the key components of good communication, and the second column describes the corresponding behavior. Row 1: Be accurate, Learn the facts about dementia, Avoid using jargon, as I might misunderstand. Row 2: Be respectful, Understand what dementia means to me, Use language preferred by me. Row 3: Be inclusive, Please talk with me, not my carer, family member or friend, Make a connection with me, Make eye contact, Use my name so I know you’re talking to me, Make sure I am listening. Row 4: Be empowering, Reduce noise and other distractions to help me to focus, Use short sentences, with one idea at a time, Be patient and understanding, Don’t rush me. Give me more time to respond. Give me time to ask questions. Row 5: Be non-stigmatizing, Do not use demeaning and derogatory terms to describe people living with dementia, Use language that recognizes me as a person and retains my feelings of self-worth.

Navigate back to Table 1..

## Table 2. Long description

A table summarizing barriers and enablers for good communication by general practice nurses with dementia patients. The table has two columns and five rows. The first column lists behaviors to operationalize selected practice recommendations, and the second column lists identified barriers and enablers. Row 1: Be accurate when talking about dementia. GPNs want dementia to be documented by the GP in the patient record before they feel they can have a direct conversation about dementia with a patient and or family. Poor knowledge about dementia presentation and impact is a barrier to good communication. Row 2: Be respectful of the person’s understanding of dementia. A lack of understanding of the different cultural contexts of dementia can be a barrier to good communication. A lack of communication skills can be a barrier, for example, skills needed to adjust language to meet the person’s level of understanding as dementia progresses. Row 3: Be inclusive and allow people the opportunity to speak for themselves. Patients trust and feel safe with nurses and will talk with them about their concerns. Competing priorities limit time during the consultation to allow the person living with dementia to process information and respond to questions. Row 4: Be empowering by providing a comfortable relaxed environment. GPNs perceive busy clinic environments are noisy and distracting so they try get person living with dementia out quickly, so the patient does not get agitated. Nurses perceive home visits result in more informative conversations as the person living with dementia and their carer are in a familiar environment. Row 5: Be non-stigmatizing and use positive language when talking about dementia. A lack in GPN confidence talking about dementia when it is perceived as a scary topic of conversation. Reluctance to ask questions about dementia because patients and or family may become upset.

Navigate back to Table 2..

## Table 3. Long description

A table with two columns and fourteen rows. The first column is labeled ‘TDF domains’ and the second column is labeled ‘Definitions (with examples contextualized for this study)’. The table describes various domains and their definitions related to the Theoretical Domains Framework (TDF). Row 1: Knowledge, An awareness of the existence of the Dementia Language Guidelines. This can include knowledge about dementia presentation and impact or ‘procedural’ knowledge (about the key elements of how to communicate well). Row 2: Skills, The ability to communicate well with a person living with dementia, acquired through practice. Interpersonal skills are included in this domain. Row 3: Social professional role and identity, Beliefs by the GPN that the patients trust nurses which facilitates patients disclosing cognitive concerns. This domain includes the social and professional role, identity and confidence of an individual in a social or work setting. Row 4: Beliefs about capabilities, Perceived competence and belief that an individual has in oneself. For example, GPNs perceived ability in good communication skills to complete a cognitive assessment tool correctly. Row 5: Optimism, Confidence that the GP will follow-up on the information GPNs gathering having a conversation about a patient’s cognitive concerns is an example of this domain. Row 6: Beliefs about consequences, A GPN belief that because there is no treatment for dementia there is no benefit of asking questions to assess cognition. This domain includes outcome expectancies and the potential for consequences including anticipated regret. Row 7: Reinforcement, Incentives, such as remuneration for longer consultations for the GPN to have the time needed to have difficult conversations about cognition and potential dementia with patients and families. Disincentives are also be included in this domain. Row 8: Intentions, A conscious decision to perform a behaviour or a resolve to act in a certain way. For example, deciding to discuss brain health and cognitive impairment during every care plan. Row 9: Goals, Setting targets, action planning and implementation intentions to achieve an outcome. For example, the GPN setting a goal to complete dementia and communication skills training within a certain timeframe. Row 10: Memory, Attention and Decision-making Process, Remembering to talk about cognition with patients would be an example of this domain which includes decision-making, cognitive attention and information retention. Row 11: Environmental context and resources, This domain can include built environment, organizational culture and resource availability that impacts on the development of skills, abilities and independence. An example of this domain may be a lack of organizational support for GPNs to attend education and training on dementia and communication skills. Row 12: Social influences, This domain includes power dynamics, hierarchy and social supports, norms and modelling that cause a person to change their thoughts, feelings or behaviours. GPN mentoring from nurses specialized in dementia and or communication skills could be an example of this domain. Row 13: Emotion, This domain includes emotional responses, including anxiety, fear, stress and burn-out impacting on a person performing a behaviour. For example, GPN may experience anxiety about upsetting patients if they talk about dementia. Row 14: Behavioural regulation, Self-reflection on outcomes of communication approaches, to identify what was successful and those that were not, could be included in this domain. Action-planning aimed at managing or changing objectively measured actions is in this domain.

Navigate back to Table 3..

## Table 4. Long description

A table with two columns and multiple rows. The first column lists theoretical domains, and the second column describes barriers and enablers to behavior change in general practice nurses (GPN) when communicating with people living with dementia. The table includes the following rows: Row 1: Social/professional role and identity AND Environmental context and Resources AND Social influences, GPNs want dementia to be documented by the GP in the patient record before they feel they can have a direct conversation about dementia with a patient and or family. Row 2: Knowledge, Poor knowledge about dementia presentation and impact on the patient and carer(s) can be a barrier to good communication. Row 3: Beliefs about Capabilities, A lack in GPN confidence talking about dementia when it is perceived as a scary topic of conversation by the nurse. Row 4: Emotion, Reluctance to ask questions about dementia because patients and or family may become upset. Row 5: Knowledge, A lack of understanding of dementia in different cultural contexts can be a barrier to good communication. Row 6: Skills, A lack of communication skills needed to adjust language to meet the person’s level of understanding as dementia progresses. Row 7: Social influences, Patients trust and feel safe with nurses and will talk with them about their concerns [enabler]. Row 8: Environmental context and resources, Competing priorities limited time during the consultation to allow the person living with dementia to process information and respond to questions. Row 9: Environmental context and resources AND Emotion, Busy clinic environments are noisy and distracting so they try to get person living with dementia out quickly so does not get agitated.

Navigate back to Table 4..

## Table 5. Long description

A table linking behavior change techniques with determinants of change in the primary care context. The table has 7 rows and 3 columns. The columns are labeled TDF domain (determinant of change), Behavior change technique (BCT), and An example of what the behavior change technique could look like in the primary care context. Row 1: TDF domain: Social/professional role and identity, Behavior change technique: No conclusive links identified between BCTs and determinants of change within this domain, Example: Not applicable. Row 2: TDF domain: Environmental context and Resources, Behavior change technique: Social support (practical), Prompts/cues, Remove aversive stimulus, Restructuring the physical environment, Restructuring the social environment, Avoidance/reducing exposure to cues for the behaviour, Adding objects to the environment, Example: Set up a quiet room for consultations with people living with dementia. Row 3: TDF domain: Social influences, Behavior change technique: Social support (unspecified), Social support (practical), Social comparison, Information about others approval, Social reward, Example: Mentoring by GPNs experienced in communicating with people living with dementia. Row 4: TDF domain: Knowledge, Behavior change technique: Instruction on how to perform behaviour, Information about antecedents, Information about health consequences, Information about social and environmental consequences, Example: Provide Information on changes in communication experienced by people living with dementia. Row 5: TDF domain: Beliefs about capabilities, Behavior change technique: Problem-solving, Instruction on how to perform behaviour, Demonstration of behaviour, Behavioural practice/rehearsal, Graded tasks, Verbal persuasion about capability, Focus on past success, Self-talk, Example: Role play real-life clinic scenarios communicating with people living with dementia. Row 6: TDF domain: Emotion, Behavior change technique: Reduce negative emotions, Example: Provide information on the benefits of a dementia diagnosis and living well with dementia. Row 7: TDF domain: Skills, Behavior change technique: Instruction on how to perform behaviour, Behavioural practice/rehearsal, Graded tasks, Example: Demonstrate good communication skills with people living with dementia.

Navigate back to Table 5..
